# Correlation of Significantly Decreased Serum Circulating Mesencephalic Astrocyte-Derived Neurotrophic Factor Level With an Increased Risk of Future Cardiovascular Disease in Adult Patients With Growth Hormone Deficiency

**DOI:** 10.3389/fendo.2021.671126

**Published:** 2021-06-16

**Authors:** Ziyu Ren, Yunting Wang, Qing Chen, Jiangchuan Long, Rui Zhang, Xun Wu, Wenjie Qian, Yue Chen, Dongfang Liu, Wei Ren

**Affiliations:** ^1^ Department of Endocrinology and Metabolism, The Second Affiliated Hospital of Chongqing Medical University, Chongqing, China; ^2^ Department of Endocrinology and Metabolism, The First Affiliated Hospital of Chongqing Medical University, Chongqing, China; ^3^ General Practice, The 958 Hospital of the People’s Liberation Army, Chongqing, China

**Keywords:** adult growth hormone deficiency, mesencephalic astrocyte-derived neurotrophic factor, insulin resistance, lipid metabolism, cardiovascular risk

## Abstract

**Objective:**

Adult growth hormone deficiency (AGHD) is a rare chronic inflammatory disease caused by damage to the pituitary gland and is accompanied by disorders of multiple metabolic pathways. By examining the correlation between the serum mesencephalic astrocyte-derived neurotrophic factor (MANF) levels of AGHD patients and those of normal controls, we hope to elucidate the close relationship among MANF, lipid metabolism and insulin resistance in AGHD and discuss the potential therapeutic value of MANF.

**Methods:**

This study included 101 AGHD patients and 100 healthy subjects matched for sex, age, height, and weight. Anthropometric parameters and biochemical indicators such as body mass index, waist circumference, hip circumference, serum MANF level, blood lipids and insulin level were measured. The above patients were also divided into several subgroups for correlation analysis based on indicators such as insulin resistance and BMI.

**Results:**

The serum circulating MANF content of AGHD patients was significantly lower than that of the normal control group (5.235 (0.507-17.62) ng/ml (n=101) *vs.* 10.30 (1.84-16.65) ng/ml (n=100); p<0.0001), and circulating MANF levels were linearly correlated with HOMA-IR in the AGHD population (R=0.481, P=0.0041). When MANF was at pathological concentrations (lower than the mean circulating MANF of normal controls), the lowest concentration tertile (OR=21.429 p<0.0001) had a significantly higher disease odds ratio, Framingham risk score and 10-year risk of atherosclerotic cardiovascular disease than the highest concentration tertile.

**Conclusions:**

MANF has a significant correlation with insulin resistance in the AGHD state. There is a strong correlation with abnormal glucose and lipid metabolism in the obese AGHD population. MANF is also a good assessment factor for the risk of cardiovascular disease in AGHD patients and has excellent therapeutic potential.

## Introduction

Growth hormone is a polypeptide secreted by somatotroph cells of the anterior pituitary gland that is released in pulses mainly at night, and it can participate in anabolism and promote the growth and development of the body (1, 2). Acquired adult growth hormone deficiency (AGHD) is a special endocrine condition in which the growth hormone secretion ability of somatotroph cells is inhibited due to structural damage to the pituitary gland in adults, ultimately resulting in lower than normal absolute levels of growth hormone. Due to the presence and widespread expression of growth hormone receptors in various tissues and organs of the body, AGHD is characterized by abnormal body composition, imbalanced energy metabolism, decreased exercise capacity, impaired heart and kidney function, and even impaired psychological health (3). It has been shown that AGHD, similar to metabolic syndrome, is strongly associated with a chronic inflammatory state and that serum levels of lipocalin-2 (LCN2) are significantly elevated in patients with AGHD compared to the healthy population (4). Numerous studies suggest that disruption of the balance of the growth hormone/insulin-like growth factor 1 axis can induce the development of various heart diseases. Patients with AGHD have elevated levels of circulating inflammatory factors, accompanied by increased levels of oxidative stress and endothelial dysfunction (5). A body of evidence suggests that patients with AGHD have a higher risk of developing cardiovascular disease.

Mesencephalic astrocyte-derived neurotrophic factor (MANF) was first discovered by Canadian scientists in 2003 as a secreted protein with selective protective effects on dopamine neurons (6). However, in recent years, researchers have increasingly turned their attention to the association of MANF with metabolic diseases. In addition to neuronal tissues, MANF protein and mRNA are also widely expressed in metabolically active nonneuronal tissues and organs such as the testis, thyroid, and adrenal gland (7). It is also significant for neuroendocrine organs such as the thalamus and pituitary gland. MANF-deficient mice have a smaller anterior pituitary gland size than wild-type mice, thus reducing the number of cells producing growth hormone and prolactin due to the reduced pituitary gland size. This eventually leads to dysregulation of pituitary hormone expression and increased endoplasmic reticulum (ER) stress and apoptosis (8). Although MANF does not share any protein sequence homology with typical neurotrophic factors, MANF can exert the same extracellular effects as typical neurotrophic factors in regulating the cellular cascade. Interestingly, MANF can also act intracellularly as a reactive protein to ER stress and participate in the unfolded protein response (9). It is well known that to balance the harmful effects of ERs, the body initiates the unfolded protein response (UPR), which stimulates the secretion of MANF to reduce the accumulation of misfolded proteins and restore the normal function of the endoplasmic reticulum (10).

Pituitary cells are more susceptible to endoplasmic reticulum stress due to their high secretion of growth hormone and various prohormones (11). Increasing evidence also suggests a strong link between MANF and pituitary cells. The MANF-/- mouse model exhibited severe growth defects compared to normal controls. The size of the pituitary gland was significantly smaller, and ER stress and apoptosis were significantly increased in the pituitary gland (8). MANF has been shown to play an important therapeutic role in a variety of endoplasmic reticulum stress-related diseases. MANF has shown promising protective effects in neurodegenerative diseases, diabetes, and ischemic diseases of the heart and brain, and it can even modulate inflammatory factor expression to suppress chronic inflammatory diseases (12).

A growing body of evidence suggests that MANF appears to have highly valuable therapeutic potential for AGHD, a specific chronic metabolic endoplasmic reticulum stress-related disorder. Whether MANF can be a novel therapeutic target for AGHD needs to be supported by various lines of evidence. The purpose of this research was to investigate whether circulating MANF is associated with newly diagnosed AGHD and to clarify the association from a clinical point of view. No relevant research about serum MANF levels in healthy subjects and patients with AGHD has been reported thus far.

## Methods

### Subjects

We recruited 101 patients (68 females, 33 males; mean age of 45.87 ± 14.45 years; range 20-76) with newly diagnosed AGHD from January 2017 to October 2020 in the Department of Endocrinology of our hospital. AGHD was diagnosed according to the insulin tolerance test (ITT), which is recommended by The American Endocrine Society as the gold standard, with a growth hormone (GH) peak <5.0 μg/L. None of the patients had been treated with GH prior to diagnosis, and all the patients were evaluated for thyroid, gonadal, and adrenal function and received stable hormone replacement therapy for more than 6 months according to the function of their respective endocrine glands to ensure the stability of these glands and related hormone levels. A total of 100 healthy subjects (66 females, 34 males; mean age of 44.71 ± 10.47 years; range 26-77) were recruited as controls for our study. The abovementioned healthy volunteers were roughly matched with AGHD patients in terms of age, sex, height, and weight. All subjects participating in this study were informed of the experimental method and purpose and provided informed consent signed by themselves. All experimental protocols conformed to the Declaration of Helsinki and were approved by the Ethics Committee of our hospital.

### Inclusion Criteria

The inclusion criteria were as follows: 1. the subjects in the experimental group met the diagnostic requirements of ITT (GH peak value < 5.0 μg/L); 2. the subjects did not have diabetes or chronic diabetic complications; 3. the subjects were not treated with drug regimens to interfere with glucose lipid metabolism; 4. the subjects did not have hypertension or cardiovascular disease; 5. the subjects did not have a mental illness or malignant tumors; 6. the subjects did not have severe or chronic kidney or liver disease; and 7. the subjects did not have acute symptoms of infection.

### Experimental Group Design

In this study, 101 patients with AGHD were divided into the following three subgroups according to different BMIs, pathogenic factors and degrees of insulin resistance to investigate the possible correlation between MANF and different factors. We first divided the 101 patients with AGHD into an overweight group (BMI>25.0 (n=34)) and a nonobese group (BMI<25.0 (n=67)) based on the World Health Organization (WHO) proposed criteria that a body mass index (BMI) over 25 is considered overweight (13). The above patients were then regrouped according to the most common cause of AGHD into a postoperative pituitary tumor group (craniopharyngioma×3; Rathke cleft cyst×2; nonfunctional pituitary adenoma ×22, n=27), an idiopathic AGHD group (menstrual disorders of unknown origin×6; primary amenorrhea×2; insidious onset×52, n=60) and a Sheehan’s syndrome group (adrenocorticotropic hormone combined with thyroid hormone deficiency×10; sex hormone combined with adrenocorticotropic hormone deficiency×4, n=14) (14). However, since all the patients with Sheehan’s syndrome were female, which may lead to gender bias in the statistical results, this study excluded all male patients in the etiological classification for subsequent comparison (postoperative pituitary tumors group (nonfunctional pituitary adenoma×19), n=19; idiopathic AGHD group (menstrual disorders of unknown origin×6; primary amenorrhea×2; insidious onset×27), n=35; Sheehan’s syndrome group (adrenocorticotropic hormone combined with thyroid hormone deficiency×10; sex hormone combined with adrenocorticotropic hormone deficiency×4), n=14). Finally, 91 patients with AGHD (10 of whom did not undergo fasting insulin testing) were divided into an insulin-resistant group (HOMA-IR > 2.71, n=34) and a noninsulin-resistant group (HOMA-IR < 2.71, n=57) according to the homeostasis model assessment proposed by the University of Oxford (15). The possible differences and associations of MANF in the above subgroups were explored separately.

### Anthropometric Parameters and Biochemical Indexes

All the participants completed a comprehensive clinical questionnaire detailing their physical measurements, including basic information on physical examination, type of nongrowth hormone replacement medication, and medication dosage. After 12 hours of fasting, all the participants had elbow venous blood collected for the evaluation of relevant biochemical parameters. Fresh serum was selected for analysis of the fasting glucose, fasting insulin (Fins), glycated hemoglobin (HbA1c), insulin-like factor-1 (IGF-1), insulin-like factor binding protein-3 (IGFBP3), circulating lipids, transaminases, and high-sensitivity C-reactive protein (hsCRP) levels. The remaining serum was promptly frozen at -80°C for the later measurement of circulating MANF levels. Plasma glucose was measured using the glucose oxidase method. Lipid metabolic spectra were measured by a biochemical autoanalyzer (CX-7 Biochemical Autoanalyzer; Beckman, Brea, CA, USA). The serum insulin, GH, and IGF1 levels were detected by chemiluminescence immunoassay (Immulite1000).

Ten-year risk scores for atherosclerotic cardiovascular disease (ASCVD) and Framingham risk scores were determined for all the participants to assess the risk level for developing cardiovascular disease over the next ten years (16, 17).

The formulas involved in this research were as follows: this assay adopts the homeostatic model to assess the insulin resistance index using the following formula: HOMA-IR = fasting insulin (mU/L) × [fasting plasma glucose (mmol/L)/22.5]. β cell function was calculated as follows: HOMA-β = [20 × fasting insulin (mU/L)]/[fasting plasma glucose (mmol/L)-3.5]. The lipid accumulation product, LAP, was calculated as follows: LAP (male) =[waist circumference, WC (cm) -65]×triglycerides, TG (mmol/L); LAP (female) =[WC (cm) -58]×TG (mmol/L). The visceral adiposity index was calculated as follows, VAI: VAI (Male) = WC (cm)/(39.68+1.88×BMI)×TG (mmol/L)/1.03×1.31/HDL (mmol/L); VAI (Female) = WC (cm)/(39.68+1.89×BMI)×TG (mmol/L)/0.81×1.52/HDL (mmol/L). The quantitative insulin sensitivity index was calculated as follows: QUICKI =1/[log(fasting insulin) + log(fasting glucose)].

### Measurements of Serum MANF

Serum circulating MANF levels were determined by human MANF ELISA kits (ab215417, Abcam, USA). The ELISA kit has an optimal measurement range of 0.25 ng/ml - 16 ng/ml and a sensitivity of 30 pg/ml. This study used the same batch of kits to avoid differences between batches that could affect the accuracy of the experiment.

### Statistical Analysis

All analyses in this study were performed with the Statistical Package for Social Sciences version 26 (SPSS Inc., Chicago, IL, USA). In this experiment, we performed Kolmogorov–Smirnov tests on all test data from participants to determine their normal distribution. Continuous variables are expressed as the mean ± standard deviation (SD), and skewed distribution data are expressed as medians with interquartile ranges. Demographic and laboratory characteristics were compared between the AGHD population and the normal control group. The Wilcoxon rank-sum test was used for comparison of skewed distribution data, and the independent samples t-test was used for normal variables. Among the subgroups divided by BMI and homeostatic model, we used Spearman’s correlation analysis to test the correlation between the MANF level and other demographic and laboratory characteristics. In multiple linear regression, we tested multicollinearity for all independent variables, and if the VIF value exceeded 5, the variables were considered to be multicollinear. After dividing all subjects by MANF concentration tertile, we used the Kruskal-Wallis H test to examine differences in Framingham risk scores and 10-year risk scores for ASCVD between groups. Binary logistic regression analysis was used to calculate the odds ratio of AGHD at different serum MANF concentrations. All reported confidence interval (CI) values were calculated at the 95% level. Categorical variables are expressed as absolute and relative (%) values or proportions. In all analyses, a P value <0.05 was considered statistically significant.

## Results

### Clinical Characteristics of the Study Subjects

All the AGHD and control subjects were matched for height, weight, age, and sex (P>0.05). **Table 1** details the comparison and differences in demographic data and laboratory parameters between the AGHD patients and healthy controls. In the AGHD group, the waist circumference and the levels of TC, TG, HDL, LDL, hsCRP, LAP, VAI, ALT and AST were significantly different from those in the control group. In the AGHD population, the circulating lipid levels were significantly increased, whereas the HDL level was significantly lower than that in the control group. The VAI, LAP, and other lipid metabolism parameters were significantly higher than those in the control participants.

**Table 1:** Main clinical characteristics in AGHD and controls.

Variables	Control (n=100)	AGHD (n=101)	P value
Age (y)	45.5(35-52)	46.00(35-56)	0.703
Height (cm)	161.14±7.19	160.09±8.43	0.329
Weight (kg)	58.621±9.84	61.59±13.52	0.411
BMI	23.71±4.01	23.03±3.46	0.501
Gender (M/F)	34/66	34/67	0.842
Waist circumference (cm)	78.08±9.11	85.48±10.94	<0.0001*
SBP (mmHg)	116.91±14.97	119.76±16.99	0.485
DBP (mmHg)	74.00(66.25-80.00)	75.00(66.25-83.00)	0.366
FPG (mmol/L)	5.1(4.90-5.40)	5.2(4.75-5.60)	0.945
TC (mmol/L)	4.44(3.87-4.75)	4.81(4.15-5.85)	<0.0001*
TG (mmol/L)	1.337±0.67	2.187±1.53	<0.0001*
HDL (mmol/L)	1.395(1.19-1.60)	1.18(0.93-1.66)	0.013*
LDL (mmol/L)	2.70(2.19-3.09)	3.09(2.30-3.60)	0.004*
hsCRP (mg/L)	0.51(0.15-0.91)	1.68(0.66-3.76)	<0.0001*
LAP	22.00(8.82-35.04)	47.12(26.79-79.66)	<0.0001*
VAI	1.525(0.91-2.47)	2.65(1.51-4.16)	<0.0001*
AST (u/L)	17.00(14.50-21.00)	22.00(18.00-30.25)	0.004*
ALT (u/L)	15.00(11.50-21.50)	21.00(14.00-30.00)	<0.0001*
ALB (g/L)	45.371±2.31	44.157±4.43	0.102
Cr (μmoI/L)	65.50(57.00-77.00)	72.00(52.25-83.75)	0.059
UA (μmol/L)	297.50(259.00-375.50)	295.00(235.00-370.00)	0.524
HOMA-IR	2.11(1.22-3.57)	1.53(0.75-2.03)	<0.0001*
HOMA-β	110.40(65.56-177.78)	58.43(38.42-107.53)	<0.0001*

SBP, systolic blood pressure; DBP, diastolic blood pressure; FPG, fasting plasma glucose; TC, total cholesterol; TG, Triglycerides; HDL, high-density lipoprotein cholesterol; LDL, low-density lipoprotein cholesterol; hsCRP, high-sensitivity C-reactive protein; LAP, lipid accumulation product; VAI, visceral adiposity index; AST, aspartate aminotransferase; ALT, alanine aminotransferase; Cr, creatinine; UA, uric acid.

The data are presented as the mean±standard deviation or medians with interquartile ranges. *The p values for the comparisons between the two groups were two-tailed and considered significant at p < 0.05.

### Serum MANF Levels in AGHD Were Significantly Lower Than Those in the Control Population

**Figure 1:**
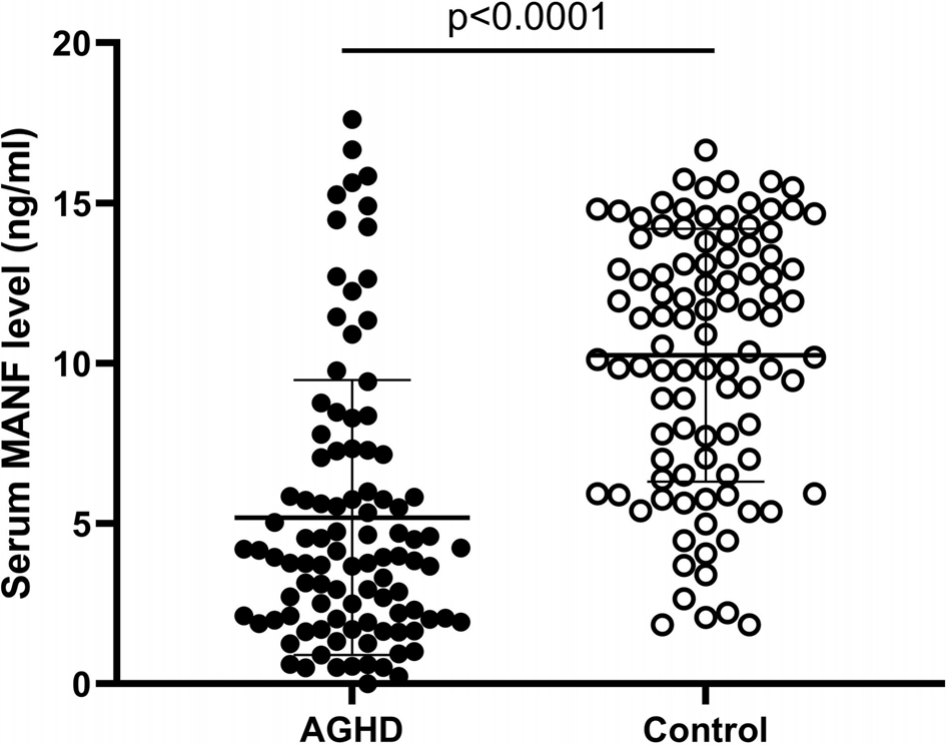
Serum circulating MANF levels in AGHD patients and control populations.

Circulating serum MANF levels were significantly lower in the AGHD subjects than in the healthy controls (5.235 (0.507-17.62) ng/ml (n=101) *vs.* 10.30 (1.84-16.65) ng/ml (n=100); p<0.0001), as shown in **Figure 1**.

### Circulating MANF Levels in Overweight AGHD Patients Were Significantly Higher Than Those in the Nonobese Group

**Figure 2:**
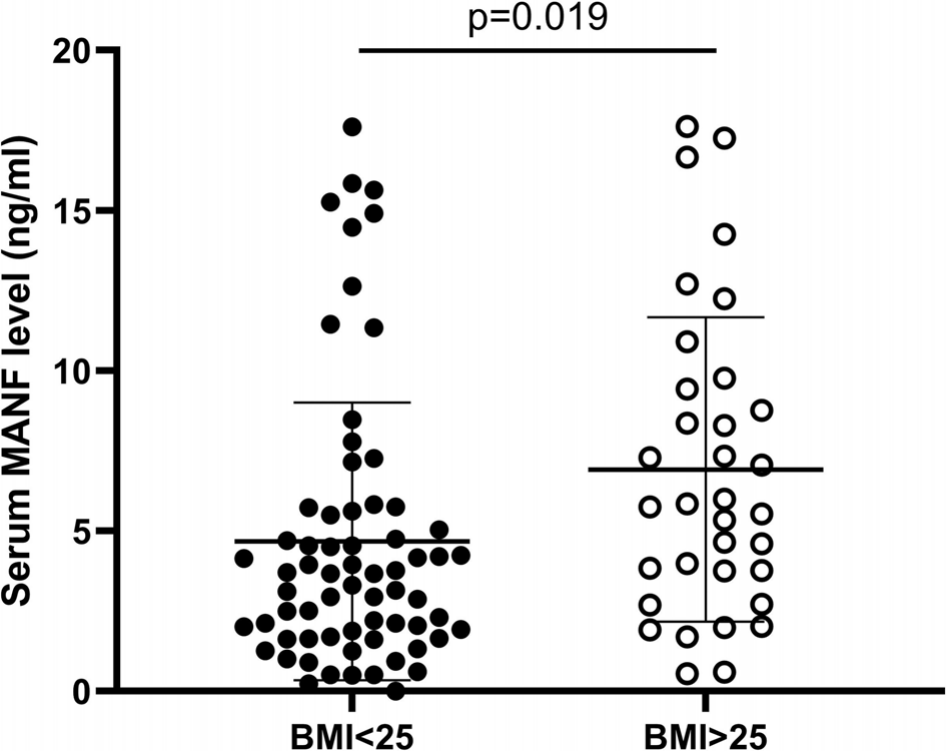
Serum circulating MANF levels in two AGHD subgroups of patients according to BMI=25.

**Figure 3:**
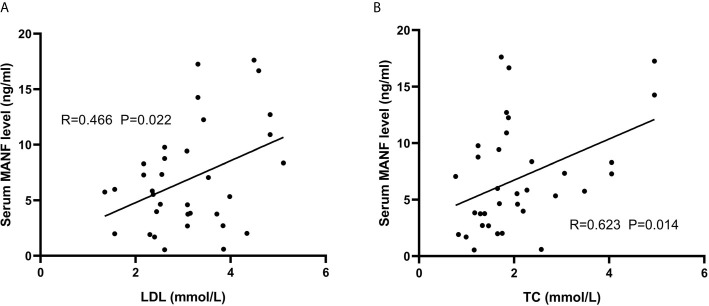
Associations between lipid metabolism indexes and circulating MANF in overweight AGHD patients. Scatter plots and correlation coefficients. Scatter plot and correlation coefficient (R) between LDL **(A)** and TC **(B)** and cycle MANF. P values were considered significant at p < 0.05.

Since AGHD is closely related to lipid metabolism, 101 patients with AGHD were grouped according to the WHO definition of BMI=25.0 as the limit of overweight, were divided into two subgroups with BMI>25.0 (n=34) and BMI<25.0 (n=67) and were subjected to comparative statistical analysis. As shown in **Figure 2**, serum circulating MANF levels in AGHD patients with BMI <25.0 were significantly lower than those in subjects with BMI >25 (4.67 (0.51-17.61) ng/ml *vs.* 6.92 (0.55-17.62) ng/ml; p=0.019). The Mann-Whitney U test suggested significant differences between the two subgroups of subjects in the Framingham risk scores, 10-year risk scores for ASCVD, Fins, HOMA-IR, and QUICK (p<0.05). All of the above data in the obese group were higher than those in the nonobese group. Next, we examined the relationship between serum MANF levels and other various parameters in patients in the BMI>25.0 group using Pearson’s correlation analysis. The results showed that serum MANF was positively correlated with the levels of TC (r=0.477** p=0.004), TG (r=0.415* p=0.015), LDL (r=0.391* p=0.022), and LAP (r=0.434* p=0.01). Multiple linear regression revealed *an independent correlation between the TC and LDL levels and the level of serum circulating MANF* ***(*** **Figure 3** *)*.

### Correlation of Circulating MANF Level and Metabolism in AGHD Patients Under Different Pathogenic Factors

Due to the diverse etiologies of AGHD, this study aimed to further investigate the association between serum circulating MANF factors and the metabolic axis in AGHD patients with different etiological factors. We categorized 101 patients with AGHD into the following three groups according to different etiologic factors: the postoperative group, idiopathic AGHD group, and Sheehan’s syndrome group, The results of the Kruskal-Wallis H test suggested that there were differences in the TC, TG, LDL, VAI, GH, and AST levels among the three groups of subjects, and thus, further comparisons were made between groups. Significant differences were found in the TC (p<0.001) and LDL (p<0.001) levels between the postoperative and idiopathic groups after Bonferroni correction (significantly higher in the postoperative group than in the remaining two groups). For comparison of the anthropometric indexes, we adjusted the sex of the patients in the postoperative and idiopathic groups and finally selected all female subjects in both groups for subsequent comparisons (group 1 n=19; group 2 n=35; group 3 n=14). In terms of height (although not reaching statistical significance, a trend can be seen, p=0.052), weight, BMI, and IGF1 level, the subjects in Sheehan’s group exhibited significantly lower values than those in the other two groups. The postoperative group exhibited significantly higher values than the other two groups in terms of the TC and LDL levels, the two lipid metabolism indexes (**Table 2**). The analysis revealed that the level of MANF was correlated with different parameters in the three subgroups. In the idiopathic AGHD group, Spearman’s correlation analysis showed that the level of MANF was positively correlated with Fins, HbA1C, VAI, QUICK, weight, BMI, waist circumference, LAP, and ALB. In the postoperative group, the level of MANF was positively correlated with DBP and UA. In conclusion, however, the level of MANF was more closely associated with lipid metabolism in AGHD.

**Table 2:** Comparison of selected parameters between the three groups of subjects after gender correction.

Pathogenic		Height,cm	Weight,kg	BMI	IGF1	TC,mmol/L	LDL,mmol/L
G1 (Postoperative, n=19)		160.28±6.31	58.72±8.51	22.31±3.36	78.85(34.40-124.75)	5.75(4.61-6.22)	3.56(2.89-3.73)
G2 (idiopathic AGHD, n=35)		156.68±6.94	57.5±11.18	23.98±4.24	121.5(83.60-221.75)	4.47(3.96-4,84)	2.42(2.02-3.14)
G3 (Sheehan's syndrome, n=14)		155.00±6.47	51.55±6.94	21.48±2.30	48.25(28.63-129.25)	4.74(4.00-5.22)	2.95(2.13-3.56)
Overall Comparison							
Intergroup comparison		0.052	0.030*	0.047*	0.002*	0.001*	0.012*
G1 VS G3	P value		0.073	0.798	1.000	0.067	0.077
G2 VS G3			0.038*	0.047*	0.025*	1.000	1.000
G1 VS G2			1.000	0.537	0.092	0.001*	0.001*

G1 means the postoperative group; G2 means the idiopathic AGHD group; G3 means the Sheehan's syndrome group. The data are presented as the mean±standard deviation or medians with interquartile ranges *The p values for the comparisons between the two groups were two-tailed and considered significant at p < 0.05.

### Circulating MANF Levels in AGHD Subjects in the Insulin-Resistant and Noninsulin-Resistant Groups

**Figure 4:**
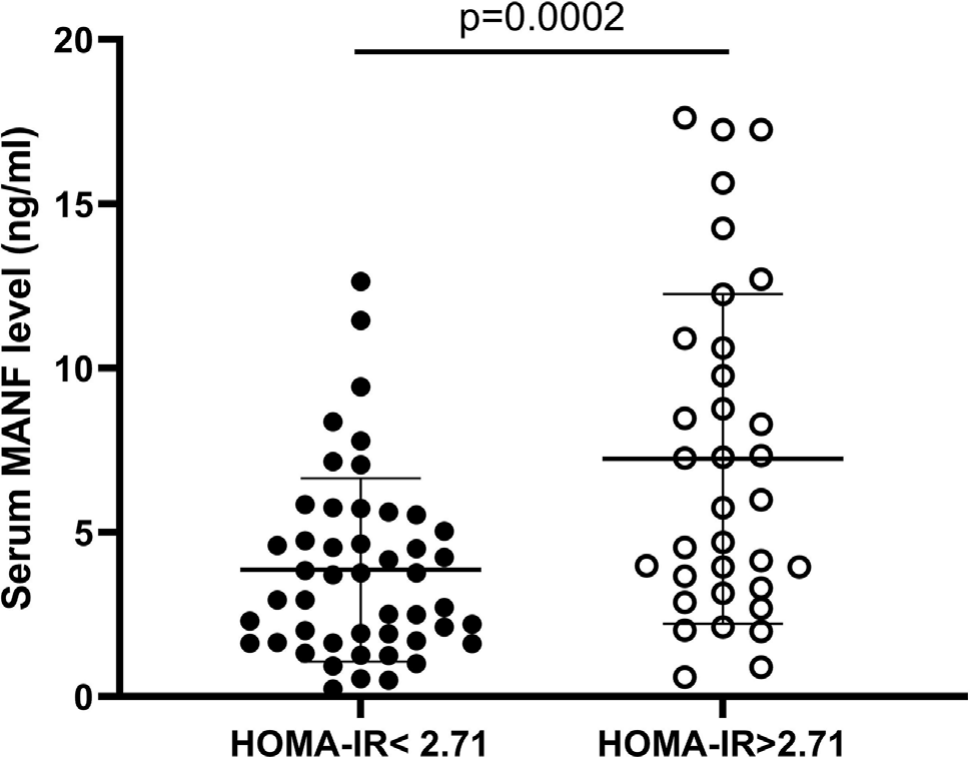
Serum circulating MANF levels in two AGHD subgroups of patients according to homeostasis model assessment.

**Figure 5:**
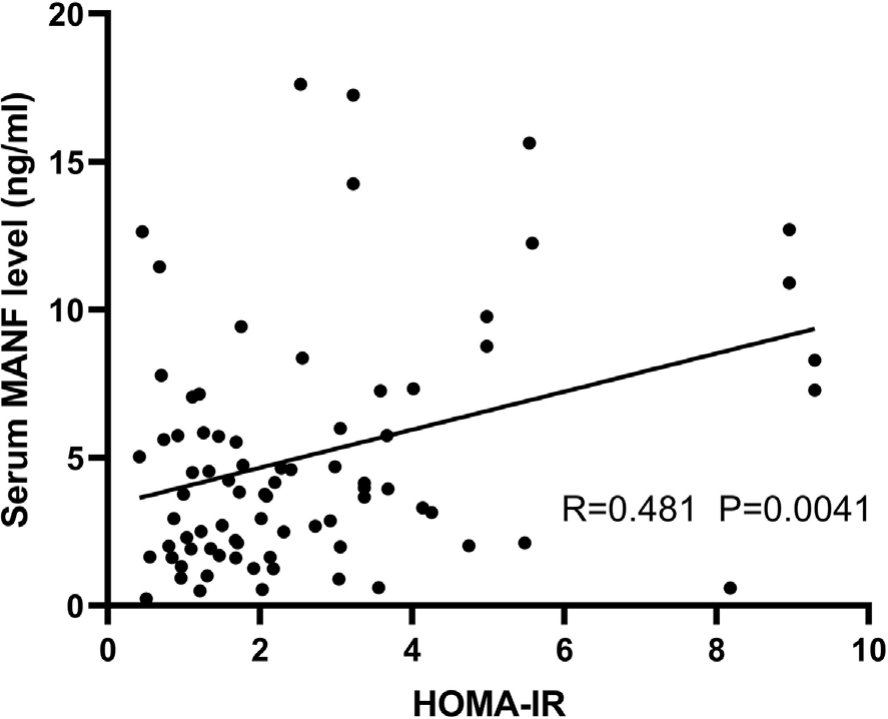
Scatter plot and correlation coefficient (R) between HOMA-IR and circulating MANF levels in the AGHD population. P values were considered significant at p < 0.05.

To investigate the relationship between circulating MANF levels and insulin resistance in the AGHD population, the circulating serum MANF levels in both groups of subjects are shown in **Figure 4**. In the insulin-resistant group, circulating MANF levels were significantly higher than those in the noninsulin-resistant group (3.86 (0.236-12.64) ng/ml (n=56) *vs.* 6.94 (0.604-17.62) ng/ml (n=34); p=0.0006). Spearman correlation analysis showed that there was a significant positive correlation between the level of MANF and Fins (r=0.306** p=0.005), HOMA-IR (r=0.288** p=0.009), HOMA-β (r=0.324** p=0.003), HbA1c (r=0.31* p=0.032), and waist circumference (r=0.331** p=0.002) in 91 subjects. To verify the existence of an independent linear correlation between MANF and insulin resistance, multiple linear regression was performed. In this experiment, *MANF level was found to have an independent influence on HOMA-IR after consecutively adding the remaining influencing factors, as shown in* **Figure 5**.

### Different Circulating MANF Concentrations and Cardiovascular Risk Assessments

We divided all the subjects into three subgroups based on the tertile of the circulating MANF concentration (group 1: <4.17 ng/ml; group 2: 4.17-10.36 ng/ml; group 3: >10.36 ng/ml). The high MANF concentration group showed lower Framingham risk scores than group 1 (p<0.0001). In terms of circulating lipid levels, group 3 also exhibited lower TC, TG, and LDL levels and higher HDL levels than group 1 and group 2 (however, due to sample size limitations, statistical significance was not reached). Binary logistic regression showed that the odds ratio for AGHD in group 1 was 20.429 times higher (OR=20.429 p<0.0001) than the ratio in group 3. The odds ratio for AGHD in group 2 was 2.869 times (OR=2.869 P=0.012) higher than in group 3. The model was adjusted for potential confounders such as height, age, sex and weight.

## Discussion

Growth hormone is involved in many metabolic pathways such as sugar, lipid, and protein pathways, but its main metabolic function is to promote lipolysis (18). Adult growth hormone deficiency is caused by structural injury to the pituitary gland or tumors in the pituitary region that leads to a sharp decrease in the amount of growth hormone in the body, which triggers structural damage to the heart, skeleton and other organs and is accompanied by endocrine metabolic disorders. An imbalance in the GH/IGF1 axis leads to an excessive accumulation of lipids, which increases the risk of cardiovascular disease. High levels of LDL as well as low levels of HDL are known to be high-risk factors for the development of cardiovascular disease (19). In this study, we found that patients with AGHD have significantly higher blood lipid levels than normal controls, which corroborates previous studies stating that AGHD patients are at a higher risk of developing cardiovascular disease (20). Interestingly, circulating serum levels of a class of endoplasmic reticulum stress-associated secreted proteins, such as MANF, were also significantly reduced in the AGHD population. To the best of our knowledge, this study is the first to link the level of MANF with AGHD. It also investigates the correlation of MANF levels with lipids, glucose metabolism and potential therapeutic value in the AGHD population.

In recent years, increasing evidence has shown that MANF has unique advantages in relation to endocrine metabolic pathways and stress protection in the endoplasmic reticulum (21). In animal models, MANF^-/-^ mice show abnormal pituitary hormone secretion accompanied by a state of insulin resistance and growth hormone deficiency, while the mice exhibit severe growth retardation and endocrine gland atrophy. The endocrine organs of MANF+/+ mice are more active and express greater levels of hormone secretion (8). However, the expression of MANF is inconsistent in human disease, and studies have shown that circulating serum MANF levels are significantly elevated in patients with primary diabetes and early diabetes with abnormal glucose tolerance (22). In another PCOS-related study, it was noted that the circulating serum MANF level of the PCOS population was significantly lower than that of the control group (23). In our study, a trend toward significantly lower circulating MANF levels was found in the AGHD population.

In response to endoplasmic reticulum stress, cells initiate the unfolded protein response (UPR) to balance the endoplasmic reticulum. The UPR has been confirmed to form the basis of chronic metabolic diseases together with inflammation, lipid metabolism and energy control pathways (24). MANF is a class of endoplasmic reticulum stress-activated protective protein factors that stimulate MANF secretion to maintain endoplasmic reticulum homeostasis in response to cellular activation of the UPR (25). According to the literature, AGHD is a special endocrine condition closely related to lipid and glucose metabolism, and its circulating MANF concentration seems to be secreted due to the activation of the UPR to play the role of endoplasmic reticulum balance. However, interestingly, our study found the opposite, a decreasing trend in MANF levels. We speculate that AGHD patients may be in a decompensated state of MANF secretion due to prolonged low levels of growth hormone stimulation, resulting in lower overall circulating levels.

Adipose tissue is an important target for growth hormone action due to the presence of GH receptors, which are extremely sensitive to the stimulatory feedback of growth hormone (26). Obesity and overnutrition induce chronic ER stress in the liver and some other tissues (27). In the present study, significant differences in MANF levels were observed in the nonobese group and in overweight patients with AGHD. Using the WHO recommended BMI=25, serum levels were significantly higher in the obese group of AGHD patients (n=34) than in the nonobese group (n=67). Circulating MANF levels in the obese group were positively correlated with the body weight, TC, Tg, LAP, VAI, and other parameters. The levels of TC and LDL were independently linearly correlated with the level of MANF. This result reflects the close relationship between MANF and lipid metabolism. Several epidemiological trials have validated the LAP and VAI as reliable markers for predicting cardiovascular disease risk in the general population (28–30). Due to the limitation of the sample size, the linear correlation between MANF and LAP, VAI, etc., was not observed in the present study, which needs to be investigated by subsequent case expansion. However, this does not preclude the use of MANF as an independent diagnostic factor in the AGHD population and as an indicator for assessing the risk of cardiovascular morbidity in obese patients with AGHD.

It has previously been shown that MANF triggers insulin resistance by enhancing the activity of phosphatidylinositol 5-phosphate 4-kinase type-2 beta (PIP4k2b, a kinase known to regulate insulin signaling) localized to the endoplasmic reticulum (31). Patients with AGHD usually show varying degrees of insulin resistance. Thus, we divided 101 patients into insulin-resistant and noninsulin-resistant groups according to the HOMA model. Likewise, there was a surprisingly significant difference in MANF levels between the two groups. MANF was significantly higher in the 34 patients with HOMA-IR>2.71 than in the noninsulin-resistant group, and the TG, waist circumference, hip circumference, waist-to-hip ratio, LAP, and VAI values were also higher in that group than in the noninsulin-resistant group. The most significant positive correlation was between the level of MANF and insulin resistance. Both visceral obesity and insulin resistance increase cardiometabolic disease risk. A 2017 study of a large sample of European populations indicated that the VAI was independently associated with an increased 10-year risk of cardiovascular disease (32). Dysregulation of sympathetic nervous and renin-angiotensin systems resulting in enhanced stimulation of both adrenergic and angiotensin II receptors is a typical feature of heart failure and hypertension and is involved in the pathogenesis of insulin resistance. Angiotensin II acts through the angiotensin receptor to inhibit the actions of insulin in vascular tissue, in part, by interfering with insulin signaling through phosphatidylinositol 3-kinase and downstream protein kinase B signaling pathways *via* generation of reactive oxygen species by nicotinamide adenine dinucleotide phosphate oxidase (33). Evidence suggests that AGHD patients with insulin resistance may have a higher risk of cardiovascular morbidity and that MANF may be a good predictor for cardiovascular risk assessment in AGHD patients.

The main manifestation of AGHD is a decrease in GH secretion, which is accompanied by a decrease in IGF1 and IGFBP3. Regrettably, we have not yet detected the correlation between MANF and IGF1 and IGFBP3. This may be because in the AGHD population, IGF1 levels do not absolutely correspond to the disease condition, and many patients may have a normal level of IGF1. It has also been shown that GH can function independently of IGF1 (34). There are many reasons why there may not be a linear relationship between MANF and IGF1. However, it is still necessary to expand the sample size to confirm this speculation in the future.

MANF has also shown good cardioprotective effects in several studies. In infarction and localized ischemic disease, MANF is secreted in large quantities after sarcoplasmic reticulum/endoplasmic reticulum calcium homeostasis is disrupted to prevent ischemic myocardial injury and has an antihypertrophic effect (35). Other types of secreted proteins do not exhibit such secretory characteristics and efficacy (36). Due to varying degrees of insulin resistance status, abnormal lipid metabolism and visceral adipose deposition lead to vascular endothelial damage, and patients with AGHD have a higher risk of cardiovascular disease (37). MANF exhibits excellent therapeutic potential due to its unique endoplasmic reticulum balancing function.

These results leave much space for investigation. The circulating MANF concentrations in the normal population were significantly higher than those in the AGHD population, which seems to suggest that MANF at physiological concentrations is a protective factor for the body and has a balancing effect on normal lipid and glucose metabolic pathways. However, as a secreted protein, MANF exerts not only an extracellular effect on the circulating paracrine pathway but also intracellular effects of binding to transmembrane receptors to regulate the intracellular signaling cascade (38). Two different modes of action also lead to the possibility that MANF may have different biological efficacies. In the present study, lower tertile MANF concentrations had the highest odds ratio for disease, whereas in the AGHD population, higher MANF was positively associated with insulin resistance and lipid deposition. We speculate that the level of MANF content is a relative concept and that under pathological concentration conditions, relatively high MANF levels instead show negative physiological effects. It has been shown that MANF interacts with PIP4k2b and triggers insulin resistance *via* an unknown pathway other than the inflammatory activation state (39). However, the background of this experiment is that MANF transgenic mice overexpress MANF factor in the hypothalamus and exhibit insulin resistance. However, no further studies have been conducted on a pathological state model with a low dose of MANF. This is the next step in our team’s research. We observed that at physiological concentrations, MANF did not differ significantly based on insulin resistance or obesity status.

The different causative factors of AGHD also provided some interesting results. The height, weight, and BMI of the patients in Sheehan’s group were significantly lower than those of the AGHD patients with the remaining causative factors. Patients with AGHD due to surgical damage to the pituitary gland had significantly higher levels of TC, LDL, and other parameters than idiopathic patients. However, the exact cause could not be explained yet. This may be due to the limitation of the specimen size in a rare metabolic disease such as AGHD, and we also need to expand the sample size in the future to advance subsequent studies.

Overall, this study leaves us with much uncertainty and many potential research directions. However, it is undeniable that serum circulating MANF levels may be an excellent target for predicting the onset of AGHD and serve as an excellent potential therapeutic factor for cardiovascular disease in AGHD patients. MANF also needs to receive more extensive attention and research.

## Conclusion

MANF, an endoplasmic reticulum stress-secreting protein, is strongly associated with insulin resistance and abnormal lipid metabolism under AGHD conditions. This factor may be critical in the early diagnosis of AGHD and is involved in the occurrence and development of AGHD. It may have good therapeutic potential for later cardiovascular disease.

## Data Availability Statement

The raw data supporting the conclusions of this article will be made available by the authors, without undue reservation.

## Ethics Statement

The studies involving human participants were reviewed and approved by the Ethics Committee of the First affiliated hospital of Chongqing medical university. The patients/participants provided their written informed consent to participate in this study.

## Author Contributions

ZR, DL, and WR jointly conceived and designed this research. ZR and YW performed the data analysis and wrote the manuscript. QC and JL collected the medical records and biochemical data for each subject. RZ, XW, WQ, and YC conducted telephone follow-up interviews of patients and collected relevant physical information and data. DL and WR reviewed the full text and guided revisions. All authors contributed to the article and approved the submitted version.

## Funding

This research was supported by the Project of the Science and Technology Committee in Chongqing 2016 (Number: cstc2016jcyjA0025) and the National Natural Science Foundation of China (Grant No. 81370467 to DL).

## Conflict of Interest

The authors declare that the research was conducted in the absence of any commercial or financial relationships that could be construed as a potential conflict of interest.
